# Supplementary material for: Build-up of serial dependence in color working memory
Source: Sci Rep. 2020 Jul 2;10:10959. doi: 10.1038/s41598-020-67861-2 (PMC7331714; doi:10.1038/s41598-020-67861-2)
Supplement: Supplementary file 1 — Supplementary file1 (PDF 993 kb) [file 41598_2020_67861_MOESM1_ESM.pdf]

Build-up of serial dependence in color working memory

João Barbosa & Albert Compte*
Institut d'Investigacions Biomèdiques August Pi i Sunyer (IDIBAPS), Barcelona, Spain
*Corresponding author: acompte@clinic.cat

Supplementary Figures S1-S4

Supplementary Table S1

Supplementary References

Supplementary Figures

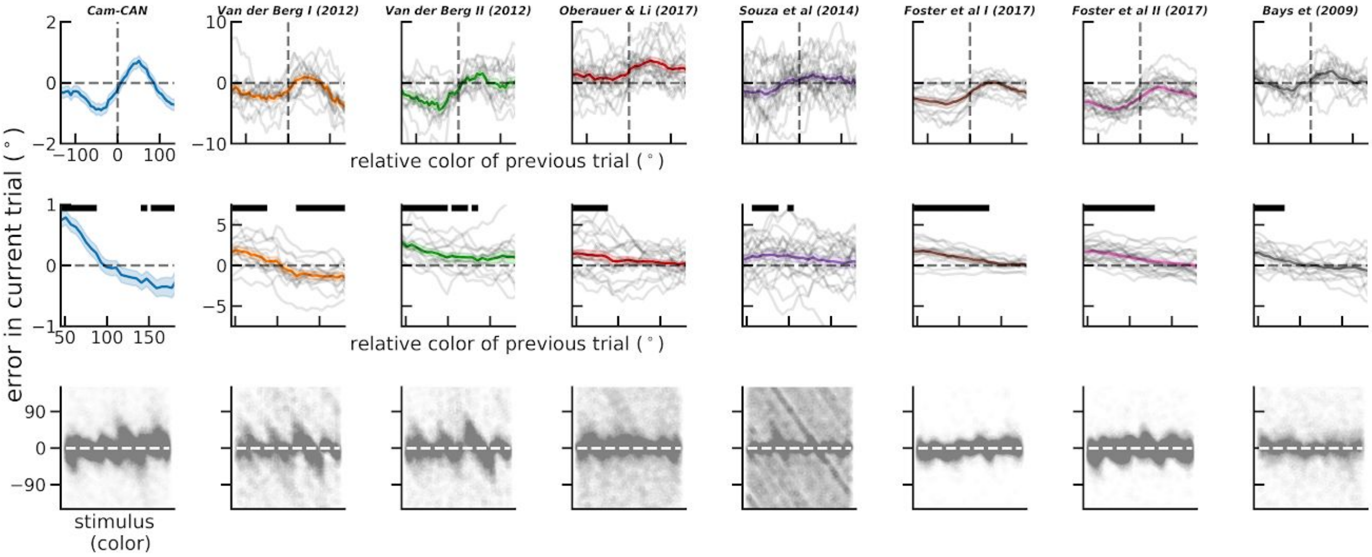

Figure S1. Serial dependence in color (folded and unfolded) for each experiment. Serial dependence in color for each experiment, unfolded at the top and folded in the middle. At the bottom, systematic errors for each experiment. For the Cam-Can dataset, we only plotted the systematic error from 100 subjects for the sake of clarity. Thick black bars on folded serial bias curves (middle) mark points where the mean was significantly different than zero (bootstrap test, p<0.05)

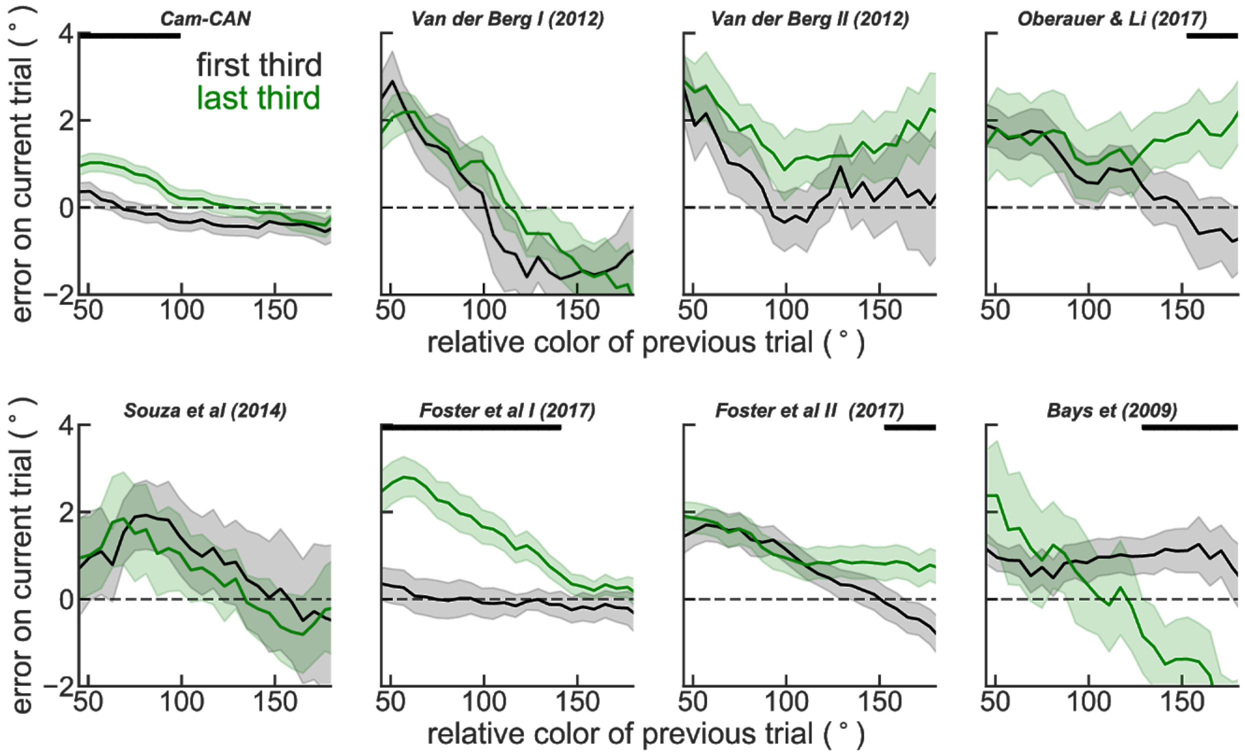

Figure S2. Serial dependence calculated using the first and the second half of each session. All error bars are bootstrapped SEM and black bars mark where the two conditions are significantly different ($p<0.05$, permutation test). Note that all studies included 12-21 subjects, except for Cam-Can's study with 649 subjects (Table S1)

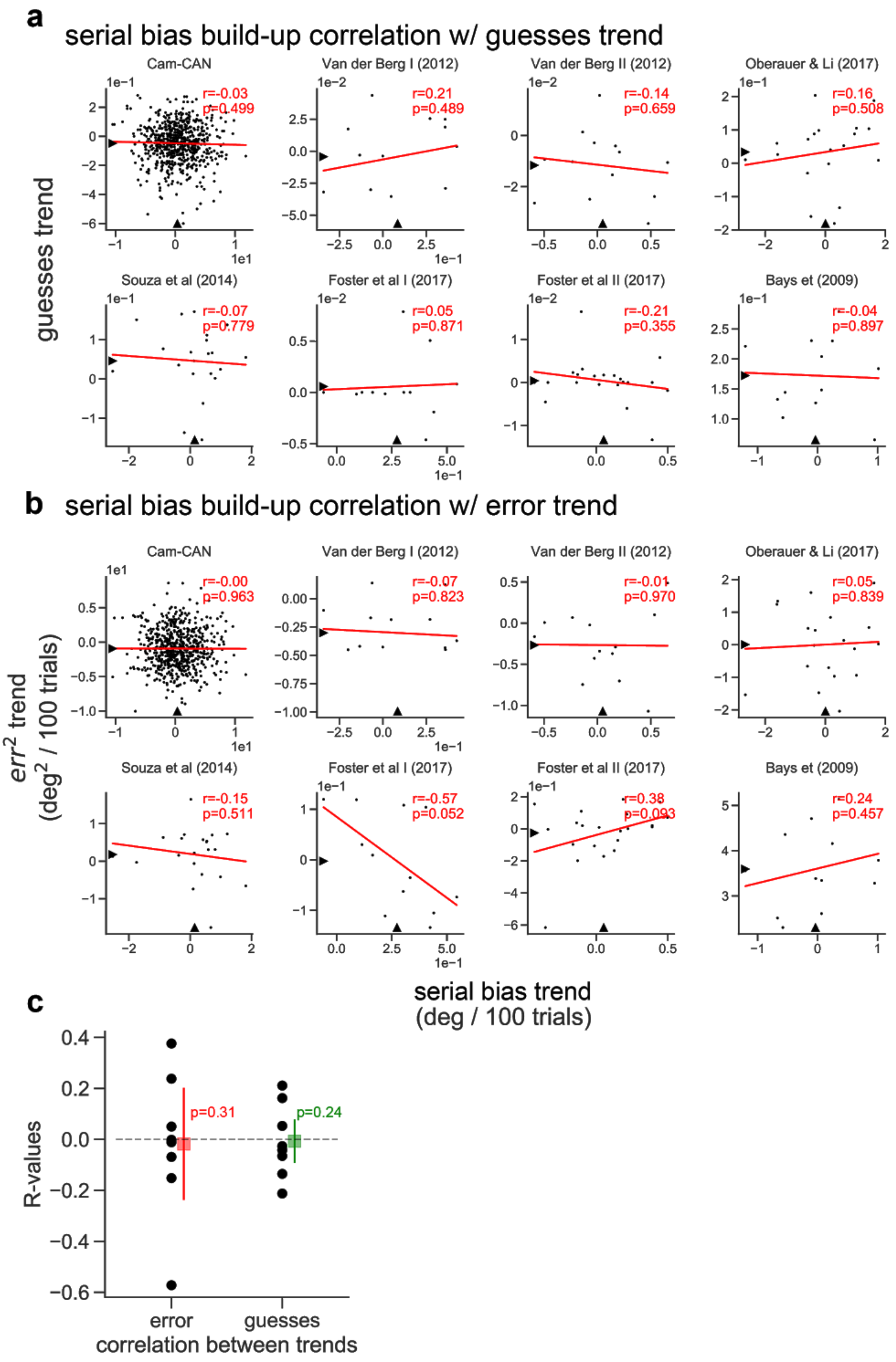

Figure S3. Serial bias build-up is independent of tiredness or task familiarity. Serial biases build-up is not correlated with trends in performance dynamics as measured by a) guesses or b) mean squared error for any dataset independently (a, b) or averaging across experiments (c). Error-bars are bootstrapped 95% C.I.

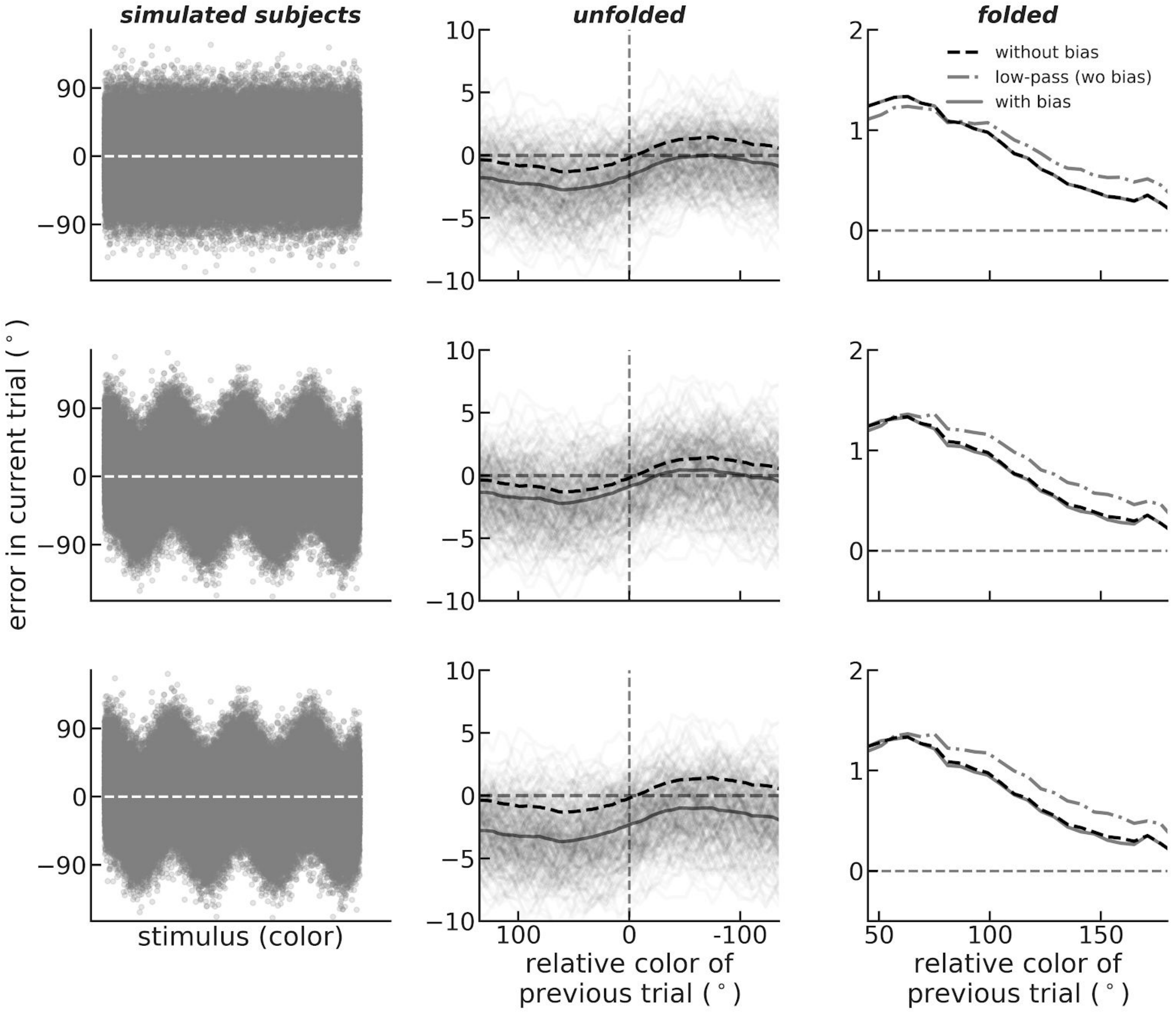

Figure S4. Same as Figure 1c, but with only systematic rotational biases **(top)**, inhomogeneities of the perceptual space **(middle)** and both **(bottom).** A folded version of serial dependence removes both systematic biases without any additional preprocessing (compare curve "with bias" with curve "without bias"). In addition, here we also plot serial dependence from the data "without bias" when removing systematic biases with a low-pass filter similar to refs. [1–3] prior to folding. Notice that the "low-pass" curve introduces biases in the estimation of serial dependence (compare to "without bias" curve).

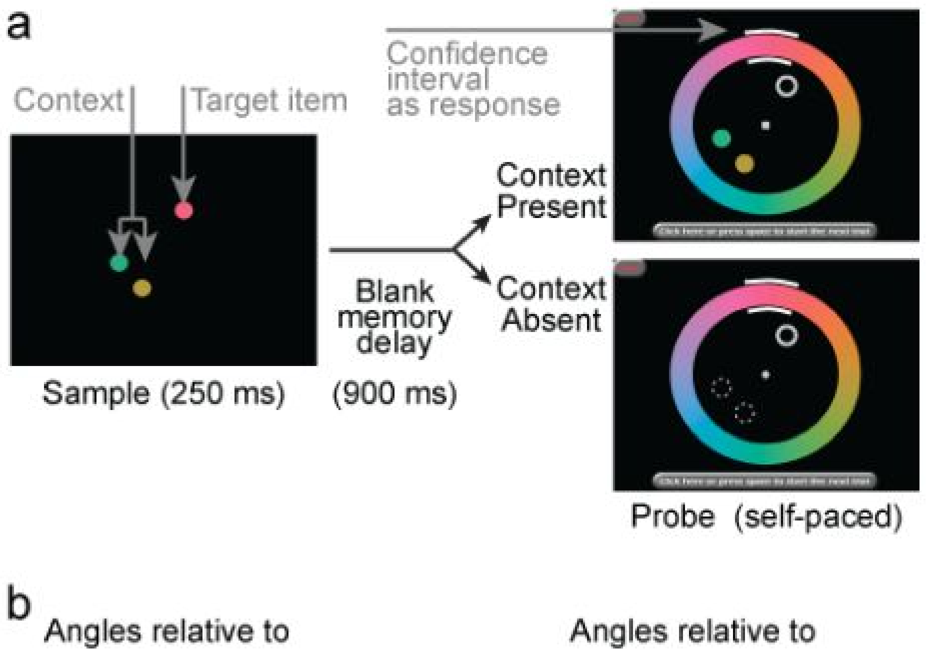

Dataset	*Set size*	*Subjects*	*Trials*	*Delay*	*Observations*
CamCan data set<br>Taylor et al (2017) [4]<br>Shafto et al (2014) [5]	1-4	649	224	0.9 s	Stimuli: circle of diameter 1.77 (dva), positions selected at random from 8 equally spaced points at an eccentricity of 4.5. 360 colors. CIE L,a,b radius of 53 and center (64,10,10). Monitor calibration procedure: none. These data was obtained from the CamCAN repository, available at: www.mrc-cbu.cam.ac.uk/datasets/camcan/<br>See for ref. [6] a thorough description of the working memory task.
Experiment 1 of<br>Oberauer & Li (2017) [7]	1-8	19	400 x 2	1 s	Stimuli: colored squares of 1.25º at viewing distance of 50cm. 360 different colors on a color wheel: CIE L,a,b = (70,20,30). Monitor calibration procedure: none.
Experiment 1 (I) of<br>Foster et al (2017) [8]	1	12	~960	1.2 s	Stimuli: circle, 1.6º diameter, centered at 3.8º at viewing distance of 100cm. 360 colors. Color wheel in Figure 1. Monitor calibration procedure: none.
Experiment 2a (II) of<br>Foster et al (2017) [8]	1	21	~960	1.15 s	Stimuli as I, plus: During presentation, a distractor with different shape and color was present at another location. Monitor calibration procedure: none.
Experiment 1 (I) of<br>Van den Berg et al (2012) [9]	1-8	13	288 x 3	1 s	Stimuli: circle, 2º diameter, centered at 4.5º at viewing distance of 60cm. 180 different colors on a color wheel: CIE 1979, L,a,b = (70,10,10). Monitor calibration procedure: none.
Experiment 3 (II) of<br>Van den Berg et al (2012) [9]	1-8	13	288 x 3	1 s	Same as I, but report done by scrolling through all possible colors (drawn uniformly and independently from the wheel). Monitor calibration procedure: none.

Experiment 2 of Souza et al (2014) [10]	1-8	21	496 x 2	1 s	Stimuli: circles of 1.1 cm diameter at 5.5cm from fixation. 360 different colors samples from hue dimension of HSL (saturation=1, lightness=.5). Condition 1: color wheel present during delay Condition 2: Last 1 sec of - sec delay was location cued. Monitor calibration procedure: none.
Bays et al (2009) [11]	1-8 (in blocks)	12	600	0.9 s	Stimuli: 2x2º patches at viewing distance of 57cm. 180 different colors samples from CIE L,a,b=(70,20,38). Presentation duration varied: 0.1, 0.5, or 2 s in a block design. This could potentially confound build-up analysis. Monitor calibration procedure: not specified.

Table S1. Experimental details of each dataset. With the exception of Foster et al I & II [8], all datasets have varying set size.

Supplementary References

Bibliography

1. Bliss, D. P., Sun, J. J. & D’Esposito, M. Serial dependence is absent at the time of perception but increases in visual working memory. *Sci. Rep.* **7,** 14739 (2017).
2. Papadimitriou, C., Ferdoash, A. & Snyder, L. H. Ghosts in the machine: memory interference from the previous trial. *J. Neurophysiol.* **113,** 567–577 (2015).
3. Papadimitriou, C., White, R. L. & Snyder, L. H. Ghosts in the Machine II: Neural Correlates of Memory Interference from the Previous Trial. *Cereb. Cortex* **27,** 2513–2527 (2017).
4. Taylor, J. R. *et al.* The Cambridge Centre for Ageing and Neuroscience (Cam-CAN) data repository: Structural and functional MRI, MEG, and cognitive data from a cross-sectional adult lifespan sample. *Neuroimage* **144,** 262–269 (2017).
5. Shafto, M. A. *et al.* The Cambridge Centre for Ageing and Neuroscience (Cam-CAN) study protocol: a cross-sectional, lifespan, multidisciplinary examination of healthy cognitive ageing. *BMC Neurol.* **14,** 204 (2014).
6. Mitchell, D. J., Cusack, R. & Cam-CAN. Visual short-term memory through the lifespan: Preserved benefits of context and metacognition. *Psychol. Aging* **33,** 841–854 (2018).
7. Oberauer, K. & Lin, H.-Y. An interference model of visual working memory. *Psychol. Rev.* **124,** 21–59 (2017).
8. Foster, J. J., Bsales, E. M., Jaffe, R. J. & Awh, E. Alpha-Band Activity Reveals Spontaneous Representations of Spatial Position in Visual Working Memory. *Curr. Biol.* **27,** 3216-3223.e6 (2017).
9. van den Berg, R., Shin, H., Chou, W.-C., George, R. & Ma, W. J. Variability in encoding precision accounts for visual short-term memory limitations. *Proc Natl Acad Sci USA* **109,** 8780–8785 (2012).
10. Souza, A. S., Rerko, L., Lin, H.-Y. & Oberauer, K. Focused attention improves working memory: implications for flexible-resource and discrete-capacity models. *Atten. Percept. Psychophys.* **76,** 2080–2102 (2014).
11. Bays, P. M., Catalao, R. F. G. & Husain, M. The precision of visual working memory is set by allocation of a shared resource. *J. Vis.* **9,** 7.1-11 (2009).
